# The role of the gut microbiome in sex differences in arterial pressure

**DOI:** 10.1186/s13293-019-0236-8

**Published:** 2019-04-25

**Authors:** Anna L. Beale, David M. Kaye, Francine Z. Marques

**Affiliations:** 1 Heart Failure Research Group, Baker Heart and Diabetes Institute, Melbourne, VIC 3004 Australia; 2 Central Clinical School, Faculty of Medicine Nursing and Health Sciences, Monash University, Melbourne, Australia; 3 Heart Centre, Alfred Hospital, Melbourne, Australia; 4 School of Biological Sciences, Faculty of Science, Monash University, Melbourne, Australia

**Keywords:** Sex, Gender, Gut microbiome, Gut microbiota, Hypertension, Blood pressure, Arterial stiffness, Preeclampsia

## Abstract

There has been intense interest in the role of the gut microbiome in human health and a broad range of diseases in recent years. In the context of cardiovascular disease, gut dysbiosis (defined as a change in the gut microbiome and the gut-epithelial barrier) has been linked to disturbances in blood pressure (BP) regulation. These findings build upon our understanding of the complex pathophysiology of essential hypertension. There are clear sex differences in the epidemiology of hypertension, with distinct trends in BP across the life-course in men and women. To date, a role for the gut microbiome in contributing to the sex differences in BP is yet to be clearly established. The purpose of this review is to summarise the current literature regarding how the gut microbiome differs between men and women and to investigate whether sex-determined differences in the gut microbiome influence the response to factors such as diet, obesity and inflammation. Finally, we will explore evidence for the possible interaction between sex-specific factors, including sex hormones and pregnancy, with the gut in the context of hypertension pathophysiology.

## Introduction

Intense interest has been directed towards the role of microbes that inhabit the human gastrointestinal tract in maintaining both health and disease states. With sophisticated culture-free methods, the genomes of the microbes in the intestine, termed the gut microbiome, can be characterised. This microbiome has been demonstrated to play roles in immunity, endocrine signalling and metabolism, amongst others [1]. Alterations to the gut microbiome and epithelial barrier, termed ‘dysbiosis’, have been implicated in the pathogenesis of gastrointestinal diseases such as inflammatory bowel disease, where faecal microbiota transplants have been successfully used as treatment [2]. Furthermore, alterations to the gut microbiome have been demonstrated to play a role in other inflammatory diseases such as asthma and allergy and more recently in diseases beyond the gastrointestinal tract, such as hypertension [3], which will be the focus of this review.

Hypertension is the leading single risk factor for mortality and global disease burden worldwide [4], and whilst it has been the subject of medical research for decades, its underlying pathophysiology remains complex and incompletely understood. Recognised factors include altered sympathetic nervous activity, increased activity of the classic arm of the renin-angiotensin-aldosterone system (RAAS) and renal extracellular fluid homeostasis, and endothelial dysfunction. In the last decade, it has also been proposed that the immune system and inflammation may play a role in pathophysiology of hypertension [5]. In this context, growing evidence now supports a possible role for the gut microbiome in the biological processes that trigger and maintain essential hypertension [3].

Essential hypertension displays considerable variability in its phenotypic expression and in its consequences. Amongst these, considerable sex differences in the epidemiology and drivers of hypertension [6] and cardiovascular disease more broadly [7, 8] are apparent. The recognition that sex differences exist in cardiovascular pathology and treatment responses has fostered a recognition of the importance of understanding the impact of sex on disease expression via basic and clinical studies [9]. On the basis of the foregoing, this review aims to examine the evidence for sex differences in the gut microbiome, and to explore whether this may play a role in the sexual dimorphism observed in the regulation of blood pressure (BP).

### Sex differences in hypertension

**Fig. 1:**
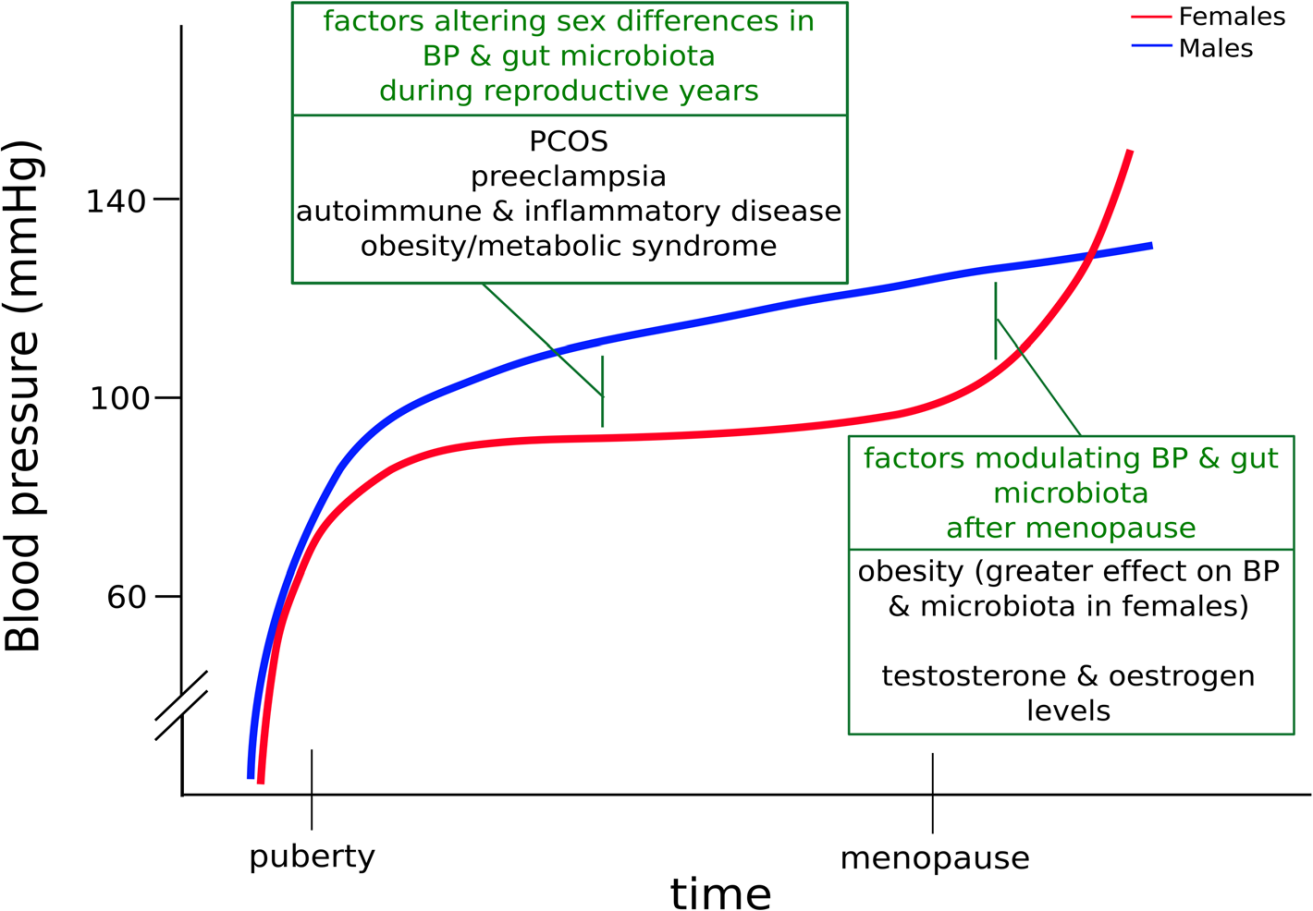
Interactions between blood pressure, sex and the microbiome across the life course. *Adapted from Colafella* et al. [6]*.* Compared to men (blue line), women (red line) are usually protected from an increase in blood pressure until they reach menopause. During reproductive years, men have higher BP than women; however, conditions such as PCOS, preeclampsia, obesity and autoimmune and inflammatory diseases, acting partly via the gut microbiome, elevate women’s BP to levels similar to, or greater than, men’s. In postmenopausal years, women’s BP increases sharply relative to men’s, driven by changes in sex hormone levels, alongside metabolic risk factors. Legend: BP, blood pressure; PCOS, polycystic ovarian syndrome

Epidemiologic studies of hypertension indicate that there are substantial sex differences in distribution (summarised in Fig. 1). The prevalence of hypertension is higher in men than in women until approximately age 65, after which the prevalence becomes higher in women (81.2 vs. 73.4% in ≥ 75-year-olds) [10]. This is accompanied by greater BP variability on ambulatory 24 h BP monitoring in elderly women than men, which conveys a higher risk of end-organ damage [11]. The higher relative prevalence of hypertension in elderly women may be partially related to premature death of hypertensive males prior to the age of 75 [6]. However, there are also substantial changes to cardio-renal mechanisms with menopause that affect women’s cardiovascular risk and prevalence of hypertension. Endogenous oestrogen has an established protective role against the development of hypertension, with effects on vasodilation that are oestrogen receptor-dependent and receptor-independent [12, 13]. These include generation of endothelium-derived nitric oxide [14], opening of calcium-activated potassium channels [15], increasing the synthesis of cyclic AMP and adenosine [16], prostacyclin production [17], and reducing the synthesis of vasoconstrictors such as angiotensin II (Ang II) [18]. Conversely, endogenous testosterone is considered to be a driver of hypertension, largely responsible for a greater rise in BP after puberty in boys compared to girls [13].

The increase in BP after menopause has a latency of 5–20 years, suggesting that factors beyond sex hormones are responsible for sex differences in the epidemiology of hypertension [13]. Differences in BP between the sexes likely stem from a complex interplay of factors, including oestrogen and testosterone, immune system and inflammatory pathways, renal function and gene expression, and are discussed in detail elsewhere [6]. There are certain factors that can modify the characteristic epidemiological trend in women, predisposing them to hypertension prior to menopause. For example, women with higher testosterone exposure, such as those with polycystic ovarian syndrome (PCOS), are more likely to develop pre-menopausal hypertension [19]. Furthermore, a number of lifestyle and environmental risk factors for hypertension have effects that are more potent in women. Smoking conveys a greater cardiovascular risk in women than men [20], which may be partially due to its effects on sex hormones [21, 22]. Obesity is also an important modifier of cardiovascular risk in women: in combination with metabolic syndrome, cardioprotection is absent in pre-menopausal women [23, 24]. For any given rise in body mass index (BMI), women have a greater rise in systolic BP than men [25]. This striking effect highlights the degree to which BP and cardiovascular risk can be altered by lifestyle factors, and how this may impact the sexes differentially.

Pregnancy may also increase the risk of hypertension in women. Gestational hypertension, defined as the onset of hypertension after 20 weeks of gestation, and preeclampsia, where there is also associated end-organ dysfunction such as proteinuria, are both associated with a substantially higher risk of hypertension and cardiovascular disease in the future [26, 27]. The effects of these pregnancy complications on the natural trend of BP are substantial; the risk of developing hypertension is 15-fold within just 2 years after a diagnosis of preeclampsia [28]. A clear understanding of the underlying pathophysiology of hypertensive disorders of pregnancy is lacking, but the maternal cardiovascular response to placental dysfunction suggests that these disorders may represent a form of maternal stress test [29].

Understanding the drivers of sex differences in BP regulation is particularly important in the context of heart failure with preserved ejection fraction (HFpEF), a disease where women are overrepresented amongst patients. Hypertension is a key player in the development of HFpEF, as it results in increased vascular stiffening, which leads to concentric remodelling and left ventricular diastolic dysfunction. These seem to be exacerbated in women [8]. Similarly, the association between elevated BP and ischaemic stroke risk is higher in women. Furthermore, hypertension is a high-risk factor for other types of cardiovascular disease where substantial sex differences are evident, such as coronary artery disease [7] and coronary microvascular dysfunction [30].

### The relationship between the gut microbiome and hypertension

Understanding the gut microbiome is made possible by sequencing the 16S ribosomal RNA gene, which has ~ 1500 nucleotides and nine hypervariable regions that differ between different bacterial taxa. Studies typically report measures of diversity of microbial bacteria: α diversity indicates the richness (number of species present in a sample) and evenness (how evenly the microbial community’s taxa are distributed) of bacteria within a sample, with greater diversity being favourable; and β-diversity refers to the distance between samples in microbial taxa. There is mounting evidence for a clear association between the gut microbiome and BP, which has been reviewed in detail previously [3, 31]. Briefly, the main evidence for the involvement of the gut microbiome in BP regulation is that (1) essential hypertensive patients have a different gut microbiome compared to individuals with normal BP [32–34]; (2) faecal transplants from hypertensive subjects to gnotobiotic (i.e. germ-free, GF) mice lead to a significant (~ 15 mmHg) increase in BP; (3) GF mice do not develop hypertension and vascular dysfunction in the presence of Ang II [35]; (4) the use of antibiotics is able to modulate BP in animal models [33, 36] and in a case-study [37]; (5) gut metabolites resultant of microbial fermentation of prebiotics such as resistant starches are cardio-protective and associated with lower BP [32, 38–40]; and (6) changes in the gut microbiome and its metabolites lead to transcriptome-wide changes in the kidney and the heart, supporting the existence of a gut-cardiorenal axis [38] and potentially of a gut-central nervous system axis [33, 36, 37, 41]. Together, these studies show that alterations to the gut microbiome and its metabolites are involved in BP regulation, by either protecting or supporting the development of hypertension.

The precise mechanisms by which the gut microbiome might influence BP, however, remain uncertain. Whilst the gut microbiome is generally very diverse with regard to bacterial species, several studies have found that it becomes less varied (i.e., α diversity, which indicates the number of species between samples, decreases) in the setting of disease. The Firmicutes to Bacteroidetes (F/B) ratio has been considered a signature of gut dysbiosis, given these are two key bacterial phyla. High prevalence of Firmicutes, resulting in a higher ratio, has been associated with a Western diet [42], and could potentially be associated with disease. However, we now know that some Firmicutes are some of the most fibrolytic bacteria, including the species *Eubacterium rectale*, *Eubacterium hallii*, *Faecalibacterium prausnitzii* and *Ruminococcus bromii* [43]. Thus, understanding the role of individual species (and perhaps communities) is likely to be more important than phyla. The microbiome can also be modulated by intake of fibre, particularly resistant starches that lower BP [38]. The mechanism involves the production of short-chain fatty acids (SCFA) such as acetate [38], butyrate [32], and propionate [39, 44] as a by-product of fermentation of fibre by intestinal bacteria.

**Fig. 2:**
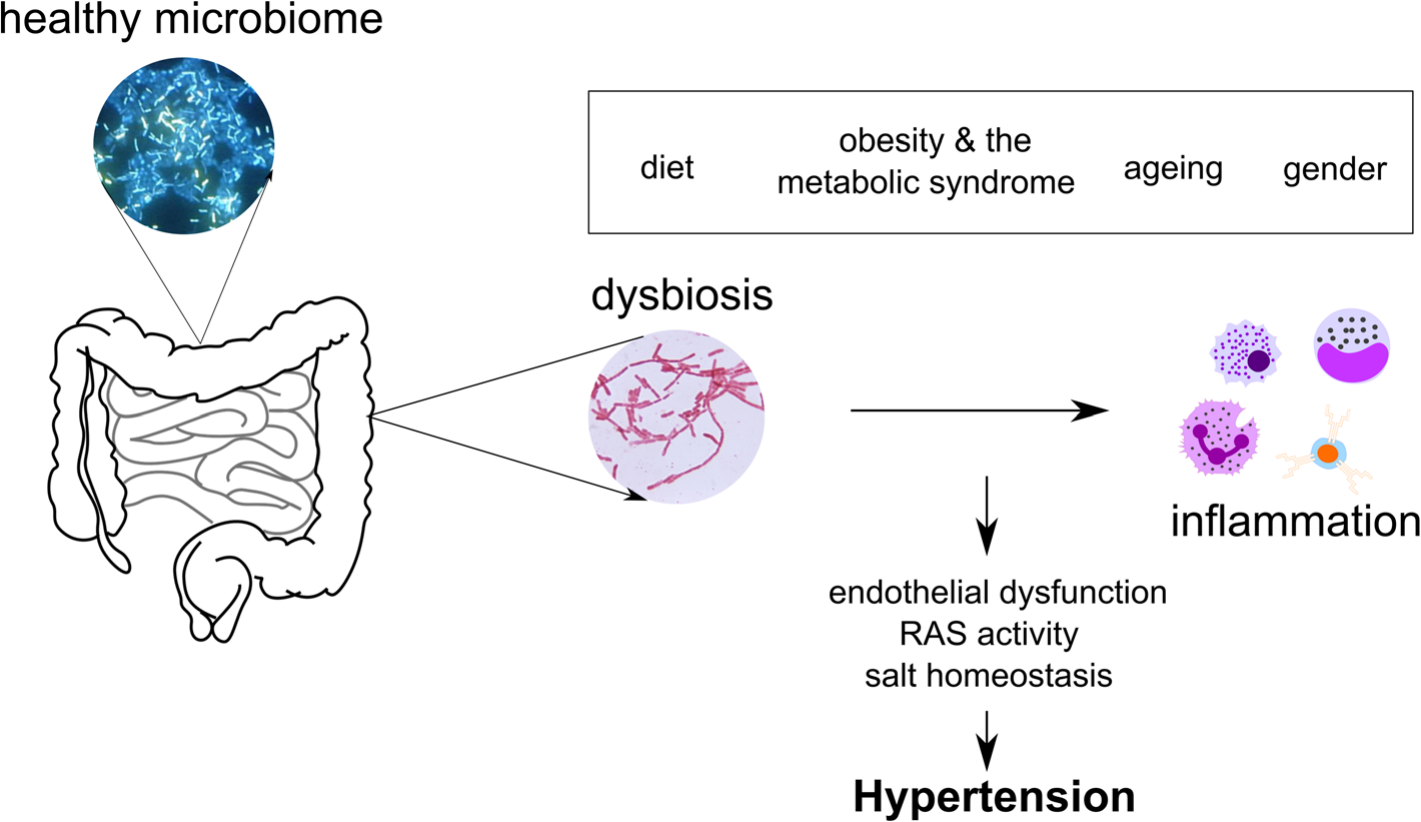
Mechanisms behind the relationship between the intestinal microbiome and hypertension. Gut dysbiosis (i.e. changes in prevalence of gut microbiota and alterations to the gut epithelial barrier) are characteristic of hypertension. This is modulated by diet, comorbidities, ageing and, likely, gender. Gut dysbiosis can lead to chronic low-grade inflammation, which can result in endothelial dysfunction, increased activity of the classic arm of the renin-angiotensin system and imbalanced salt regulation, contributing to a raise in blood pressure. Legend: RAS, renin-angiotensin system

Experimental data provides strong evidence for an interaction between the gut microbiome and BP. The possible mechanisms behind this are manifold [3], but inflammation seems to be central to this relationship (Fig. 2). For example, fibre and acetate contribute via downregulation of early growth response protein 1, which has roles in cardio-renal fibrosis, cardiac hypertrophy and inflammation; along with downregulation of interleukin-1, which is strongly pro-inflammatory, and relevant to cardiovascular disease [38]. The other SCFAs appear to have a similar anti-inflammatory roles. Inflammation has an established role in hypertension [45–47], and low-grade inflammation has also been implicated in treatment-resistant hypertension, via end-organ damage which perpetuates the hypertensive state [33]. The importance of inflammation in mediating Ang II induced hypertension and vascular dysfunction was elegantly demonstrated in the GF model: GF mice do not show high BP or inflammation when challenged with Ang II [35].

### Sex differences in the gut microbiome

Despite clear epidemiologic and pathophysiologic differences in BP control in men and women, to date, only a limited number of studies have addressed the potential interaction between sex, the gut microbiome and hypertension. However, there is some evidence to support that the gut microbiota is different according to sex. In a large cohort from four European countries, at all ages, males had higher levels of bacteria from the genera *Bacteroides* and *Prevotella* than females [48], which may reflect diet and has been demonstrated to play a role in weight loss [49].

A detailed analysis in mice explored the relationship between the gut microbiome, sex hormones and diet. Substantial sex differences in α and β diversity, both measurements that reflect gut microbial diversity, have been reported [50]. The magnitude and direction of change for multiple bacterial genera differed according to the strain of mouse, which may indicate that some of the effect of gender on the gut microbiome is mediated by interactions with the host genotype [50]. When mice were fed diets containing high levels of fat or sucrose compared to standard chow, there was clear segregation according to sex and diet [50]. This is consistent with twin studies that demonstrated that there is considerable variation between monozygotic twins, indicating a strong environmental element and a smaller contribution of the human genome to the gut microbiome [51]. Gonadectomy studies permitted examination of the effect of sex hormones, revealing that in male mice, sex hormones affected the microbiome on both standard and high-fat diets, whereas in females this effect was more marked on a high-fat diet. This builds on previous work in fish, mice and humans highlighting strong interactions between diet and sex in determining the gut microbiome [52]. Importantly, these studies highlight that sex is a relevant consideration when examining the effect of diet on the gut microbiome [50].

Also relevant to the interaction between diet, sex and the gut microbiome and their effect on hypertension is how men and women’s diets differ in a real-world setting. A study of over 200,000 adults aged 40–69 years of age from the UK has helped to characterise sex differences in dietary patterns in Caucasian western populations [53]. Women had higher energy consumption standardised by body weight, with 42% consuming more energy than recommended compared to 32% of men. Odds ratios for non-adherence to UK government dietary guidelines revealed striking gender differences: women were 2.4 times more likely to consume excess sugar, 1.4 times as likely to consume excess fat, and 1.4 times as likely to have a fibre intake below the recommendations [53]. This study highlights recent shifts in eating habits that may influence the natural history of hypertension in men and women in the near future. This has implications for the gut microbiota composition, given fibre is central to SCFA production and microbial symbiosis, whereas sugar and excess saturated fat tip the gut microbiota towards dysbiosis [54], and could contribute to sex differences in BP.

Specific dietary components have also been examined. A rat model was used to investigate the effect of oligofructose supplementation in males and females, and whether there were impacts on inflammatory parameters [55]. Oligofructose is a fructan, reaching the colon undigested, where it is metabolised by gut bacteria. It stimulates the production of SCFAs, which improve gut health, and play a role in immune function [55]. Oligofructose supplementation lead to sex differences in β diversity, but it only increased the production of SCFAs in male, and not female, mice [55]. Interestingly, oligofructose supplementation increased richness but not α diversity, and the microbiome differed more according to sex than diet. From an immune standpoint, colonic tissue cytokine concentrations, T cells and macrophage numbers were higher in females than males, and were largely unaffected by the change in diet. This is consistent with findings in the gut mucosal microenvironment in healthy men and women [56]. Pre-menopausal women had higher levels of expression of genes related to immune function and inflammation in the gut mucosa than age-matched males, with higher CD4+ T cell activation, which are associated with pre-clinical hypertension [5]. However, given women are typically protected against hypertension in pre-menopausal years, this suggests that their predisposition to gut mucosal inflammation needs to be paired with other factors, such as comorbidities and metabolic derangements to produce clinically relevant BP elevations.

Expanding on these findings, a number of studies have investigated the role of the gut microbiome in determining a predisposition of females to autoimmune diseases. This is of relevance to BP given the immune system has been implicated in the pathophysiology of hypertension [5]. Sex hormones have been shown to influence the gut microbiota in non-obese type 1 diabetic mice [57, 58]. α diversity is similar between the sexes prior to puberty; however, following puberty the bacterial families differ substantially, mainly driven by a greater deviation from the pre-pubescent microbiome in males [57, 58]. Accordingly, although female mice usually have a higher incidence of type 1 diabetes [57], GF mice have a substantial decrease in this gender bias, which seems to be mediated by the interaction between testosterone and the microbiome [57, 58]. The gender bias is also closely related to pro-inflammatory pathways involving IFN-γ and IL-1β [57], along with T cell function [58].

The relationship between testosterone and the gut microbiome is also relevant to PCOS, which is a key modifier of hypertension risk in premenopausal women. Gut microbial profiles revealed lower α diversity in 73 women with PCOS compared to 48 age-matched controls, with an intermediate phenotype in 42 women with polycystic ovarian morphology without features of hyperandrogenism or oligomenorrhoea [59]. Total testosterone level and hyperandrogenism correlated negatively with α diversity, whilst number of menses per year correlated negatively with α diversity. Interestingly, no association was observed between α diversity and age or BMI. Bacterial taxa with lower abundance in women in PCOS were all SCFA producing bacteria. Thus, whilst testosterone can be protective against autoimmune disease, elevations in testosterone in women in association with PCOS are detrimental to the gut microbial homeostasis, which may in turn affect BP.

Obesity also has established impacts on the gut microbiome [60]. Two studies in human subjects of average age 60 have addressed the relationship between obesity, sex and the gut microbiome [61, 62], with sex differences in β diversity [62], Bacteroidetes abundance [62] and certain bacterial genera [61]. A stronger relationship between BMI and gut microbiome composition was demonstrated in women than men [62], and the microbiome differed between genders in a BMI-specific manner, with higher F/B ratio in obese women than obese men [61]. This may suggest a greater role of the gut microbiome in obesity in women, given a higher F/B ratio is associated with obesity in animal models and humans alike [60], and lower Bacteroidetes abundance has been associated with obesity previously [63]. Furthermore, this may suggest a greater adverse impact of obesity in women, which is consistent with data showing greater effects of obesity on left ventricular geometry and cardiac remodelling [64]. Thus, changes in the gut microbiome with obesity in women could contribute to the loss of cardioprotection and considerable rise in BP in premenopausal women with obesity and the metabolic syndrome [23, 24].

**Fig. 3:**
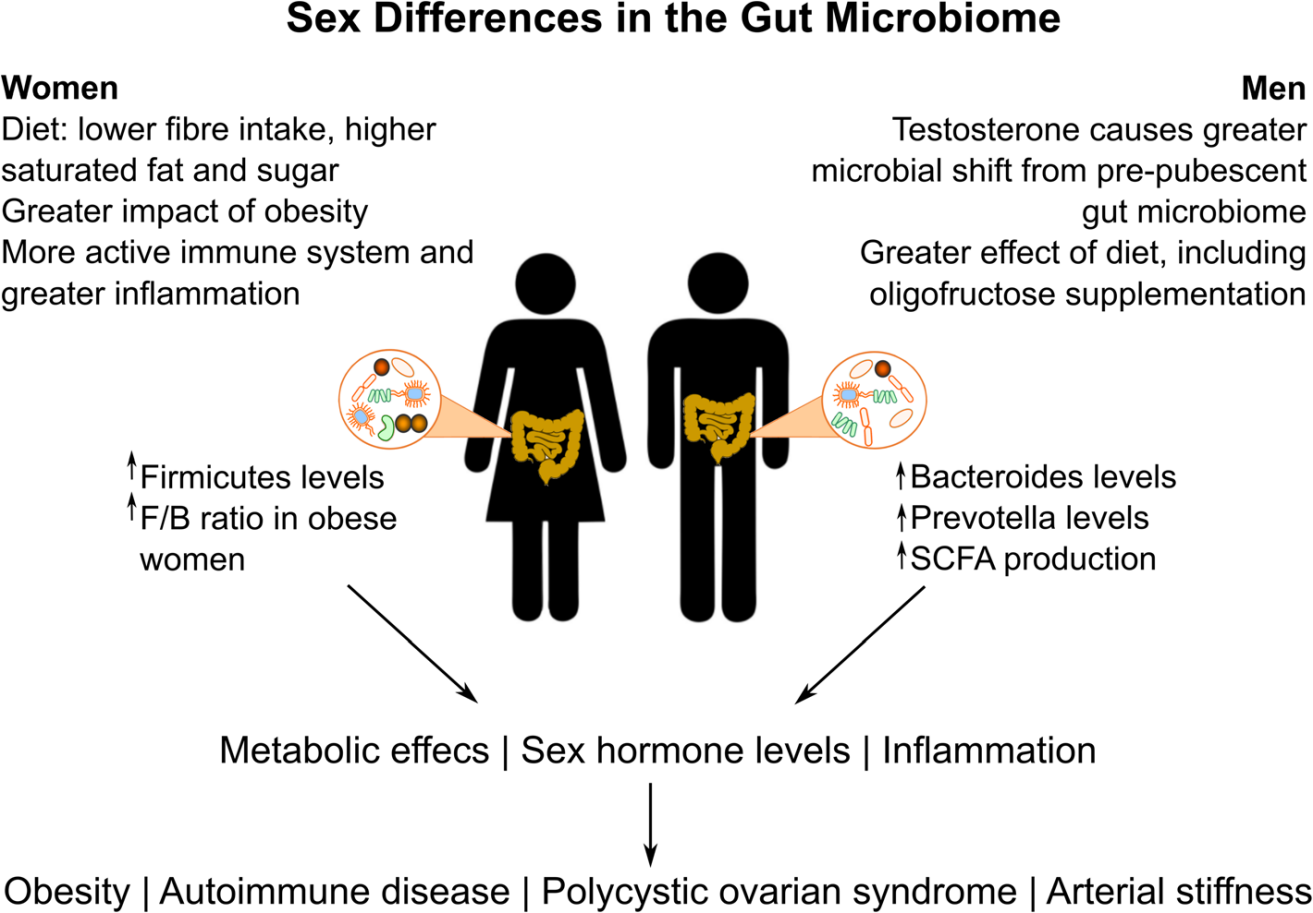
Sex differences in determinants, constituents and effects of the gut microbiome. The gut microbiome in men and women diverges after puberty, which is modulated by a range of factors, including sex hormones [57, 58], diet [50, 53] and the impact of metabolic [61, 62] and inflammatory [58] states. The resultant shifts in gut microbiome signature in turn affect inflammation, metabolism and sex hormone levels and contribute to the pathogenesis of obesity, autoimmune disease, PCOS and the development of arterial stiffness. Legend: F/B, *Firmicutes/Bacteroides*; SCFA, short-chain fatty acids

As discussed above, testosterone impacts on the gut microbiome, being largely responsible for the divergence in microbial signature between the sexes after puberty [57]. Accordingly, a decline in testosterone with ageing may adversely affect the gut microbiome in men. Changes in the gut microbiome are also able to influence testosterone levels [58], and similarly, the microbiome can influence levels of non-ovarian estrogens [65]. The contribution of the microbiome to levels of non-ovarian estrogens, which depend on deconjugation in the distal gut before re-entering the circulation via the portal system was investigated in a cohort of 25 men, 7 postmenopausal women, and 19 premenopausal women [65]. Men and postmenopausal women had similar oestrogen levels, whereas premenopausal women differed substantially [65]. When studied together, men and postmenopaual women had significant correlations between α diversity and oestrogen levels after adjusting for age, body mass index and sex [65]. These associations were not present in premenopausal women. They also demonstrated a clear relationship between low microbial diversity and enzymatic activity and greater faecal oestrogen excretion [65]. Thus, the gut microbiome is influenced by and modulates oestrogen and testosterone levels. Interactions between sex and the gut microbiome are summarised in Fig. 3.

### The role of the gut microbiome in sex differences in blood pressure

Studies directly linking the impact of the gut microbiome on sex differences in BP and hypertension are scarce. Arterial stiffness is closely related to hypertension; it is an independent predictor of cardiovascular risk and is particularly relevant to women, who see a marked rise in vascular stiffness after menopause, mirroring the rise in hypertension after menopause [66]. The decline in oestrogen clearly plays a central role in this relationship, but factors such as inflammation also contribute [67]. Furthermore, women are more sensitive to the adverse effects of arterial stiffening, with greater augmentation indices and adverse ventricular remodelling [68, 69]. The gold standard measure of arterial stiffness is carotid-femoral pulse wave velocity (PWV), indicating the speed of propagation of the arterial pulse wave through the vasculature. It consistently predicts cardiovascular outcomes in the general population and subpopulations with hypertension and diabetes mellitus [67]. There is one study to date that investigated the role of the gut microbiome in altering arterial stiffness [70]. In this study, an analysis of the gut microbiome composition was performed together with metabolomic measures and PWV in 617 female twins from the TwinsUK registry, at mean age of 61 ± 7 years [70]. They found a significant association between α diversity and PWV, persisting after adjustment for age, BMI, mean arterial pressure and family relatedness [70]. Specific bacterial taxa were also negatively correlated with PWV, including members of the *Ruminococcaceae* and *Rikenallaceae* families, which are SCFA-producing bacteria [70]. The fact that these findings persisted after adjusting for inflammation as reflected by C-reactive protein levels, smoking/alcohol habits, physical activity, fibre and omega 3 intake, Mediterranean diet adherence, socioeconomic status and proton pump inhibitor use was compelling [70]. Furthermore, visceral fat or insulin resistance did not explain these associations. Whilst these two factors explained 1.8% of variation in arterial stiffness in this cohort, gut microbial diversity and metabolites explained 8.3% [70]. This study strengthens our knowledge of the relationship between the gut microbiome, inflammation and arterial stiffness and pressure in women [70]. Repeating this study with the inclusion of a male cohort, along with premenopausal women and age-matched men would help to establish the contribution of the gut microbiome to sex differences in arterial stiffness and pressure.

### Pregnancy, blood pressure and gut microbiome

To our knowledge, there are just two studies examining differences in the gut microbiome in the context of hypertensive disorders of pregnancy. Pathogenic bacteria *Bulleidia moorei* and *Clostridium perfringens* were increased in preeclampsia in 26 women in late pregnancy compared to 74 healthy women evenly split across early, middle and late pregnancy [71]. Conversely, the beneficial bacteria *Coprococcus cactus*, which plays a role in SCFA production, was reduced in preeclampsia [71]. There were, however, no statistically significant differences in α and β diversity and abundance differed between groups. A study of 205 overweight and obese women at 16 weeks gestation investigated the relationship between the gut microbiome and BP in pregnancy, finding a negative correlation between systolic and diastolic BP and *Odoribacteraceae* and *Clostridiaceae* families, both of which are butyrate producers [72]. Furthermore, *Odoribacter* abundance correlated negatively with the inflammatory marker plasminogen activator inhibitor-1, which has increased expression in hypertensive disorders of pregnancy and preeclampsia [72]. Together, these studies are suggestive of a role for the gut microbiome and SCFAs in hypertensive disorders of pregnancy. Further research is required to better characterise this relationship and determine the use of SCFAs as therapy.

### Future directions

The hypothesis that the gut microbiome is a player in determining sex differences in arterial pressure is supported by the fact that there are clear dimorphisms in the immune system between men and women [73], and the relationship between the microbiome and immune function is substantial [74]. However, these conclusions are largely based on associative studies, and the role of the microbiome in driving sex differences in arterial pressure has not been specifically studied to date. There is significant scope for further exploration of the role of the gut microbiome in sex differences in BP, hypertension and cardiovascular risk, particularly in regards to the interaction between the microbiome and ageing in men and women including menopause (Table 1) and for the consideration of sex-specific anti-hypertensive therapies that take into account the role of the gut microbiota.

**Table 1:** Questions for further research into the role of the microbiome in mediating sex differences in blood pressure

Questions for future research	Possible implementation
The role of the gut microbiome in sex-dimorphisms in hypertension	
Is the intestinal microbiome different between hypertensive women and men?	Studies of intestinal microbial signatures in men and women with normal blood pressure, pre-hypertension and hypertension.
Does the gut microbiome change with menopause in women, and parallel changes in blood pressure?	Longitudinal studies of women starting prior to menopause.
Can HRT after menopause affect the intestinal microbiome?	Cross-sectional studies of women grouped according to HRT use.
Does the modulating effect of obesity and metabolic syndrome on blood pressure in pre-menopausal women act via the microbiome?	Studies of pre-menopausal women including cohorts of hypertensive obese and normotensive obese women.
The role of the gut microbiome in female-specific hypertensive syndromes	
What is the role of the intestinal microbiome in driving hypertension and cardiovascular complications of PCOS?	Studies of women with PCOS within and without hypertension and cardiovascular sequelae.
How does the microbiome change across the spectrum of normotensive pregnancies, gestational hypertension and preeclampsia?	Studies of women at a similar stage of pregnancy with and without these disorders.
Can the microbiome of women with PCOS or hypertensive disorders of pregnancy induce hypertension?	Animal models/germ-free experiments using microbial transfer from affected women.
How the gut microbiome impacts downstream complications of hypertension	
Is arterial stiffness greater in ageing women as a result of changes to their gut microbiome signature?	Correlation of PWV, hypertension and ventricular-vascular coupling with α and β diversity in men and women.
Does the gut microbiome play a role in determining the development of HFpEF vs. HFrEF in women vs. men?	Studies of microbial signatures in patients with HFpEF and HFrEF, stratified by gender.
Is greater ventricular remodelling and diastolic dysfunction in response to hypertension related to the microbiome in women?	Studies correlating α diversity with left ventricular geometry and haemodynamics in hypertensives and controls across both sexes.
To what extent does inflammation mediate the relationship between the microbiome, sex differences in hypertension and its complications?	Investigation of immune activation and inflammatory cytokines, and correlation with microbial signatures and complications in hypertensive men and women.

Legend: *HRT* hormone replacement therapy, *HFpEF* heart failure with preserved ejection fraction, *HFrEF* heart failure with reduced ejection fraction, *PCOS* polycystic ovarian syndrome, *PWV* pulse wave velocity

## Conclusion

Evidence continues to expand for a role of the gut microbiota in modulating essential hypertension, BP and arterial stiffness. There are bidirectional interactions between sex hormones and the gut microbiome, as well as a role for sex in the relationship between the gut microbiome and BMI, diet and immune pathways. However, how the gut microbiome modulates sex differences in BP is yet to be specifically examined, and should be the subject of further research. These should be done alongside studies to investigate whether the gut microbiome plays a role in hypertension during pregnancy, and if it has potential to be manipulated as therapy. Furthermore, whilst analysis of the bacterial 16S rRNA gene has facilitated the rapid expansion of our knowledge of the gut microbiome, future studies should investigate the role of archaea, viruses (particularly bacteriophages) and fungi in the development of hypertension, as these might also be sex-specific.

## Abbreviations

Ang II: Angiotensin II
BMI: Body mass index
BP: Blood pressure
F/B ratio: Firmicutes to Bacteroidetes ratio
GF: Germ-free
HFpEF: Heart failure with preserved ejection fraction
PCOS: Polycystic ovarian syndrome
PWV: Pulse wave velocity
RAS: Renin-angiotensin system
SCFA: Short-chain fatty acids
